# Maximizing the catheter-to-vessel size optimizes distal flow control resulting in improved revascularization in vitro for aspiration thrombectomy

**DOI:** 10.1136/neurintsurg-2021-017316

**Published:** 2021-03-15

**Authors:** Raul G Nogueira, David Ryan, Liam Mullins, John Thornton, Seán Fitzgerald

**Affiliations:** 1 Marcus Stroke & Neuroscience Center, Grady Memorial Hospital, Emory University, Atlanta, Georgia, USA; 2 Department of Mechanical Engineering, National University of Ireland Galway, Galway, Ireland; 3 Perfuze Ltd, Galway, Ireland; 4 Department of Radiology, Royal College of Surgeons Ireland, Beaumont Hospital, Dublin, Ireland; 5 Department of Physiology, National University of Ireland Galway, Galway, Ireland

**Keywords:** balloon, catheter, stroke, thrombectomy, device

## Abstract

**Background:**

Balloon guide catheters (BGCs) achieve proximal flow control during thrombectomy but antegrade intracranial flow often persists via the Circle of Willis. Closely sizing an aspiration catheter to the target vessel might achieve greater flow control and improve technical performance. Our objective was to measure the impact of aspiration catheter size on distal flow control and flow reversal with and without the use of BGCs. Clot retrieval testing was performed to establish the impact of these parameters on revascularization.

**Methods:**

An in vitro thrombectomy model replicated in vivo conditions. Flow was measured continuously using ultrasonic flow sensors placed 20 cm distal to the catheter tip in the middlel cerebral artery (MCA). Four aspiration catheters of increasing size were evaluated: ACE 60 and 64 (Penumbra), SOFIA Plus (MicroVention), and Millipede 088 (Perfuze). Two clot analog types (red blood cell-rich and fibrin/platelet-rich) were used for clot retrieval testing.

**Results:**

The larger area of the ‘superbore’ Millipede 088 catheter resulted in a larger reduction in antegrade flow than standard aspiration catheters, even when the latter were combined with a BGC. During aspiration, 6Fr catheters were unable to cause flow reversal in the distal MCA while the Millipede 088 achieved significant distal flow reversal (−146 mL/min) (P<0.0001*) (*denotes significance). The solo use of Millipede 088 resulted in better recanalization outcomes and significantly reduced distal emboli for internal carotid artery (P=0.015*) and MCA (P=0.014*) occlusions compared with all other devices and combinations.

**Conclusions:**

Maximizing the catheter-to-vessel size facilitates near flow-arrest on catheter insertion, potentially negating the need for a BGC. A 0.088 inch aspiration catheter enables significant flow reversal in the distal MCA during aspiration.

## Introduction

Balloon guide catheters (BGCs) are commonly used to achieve proximal flow control during mechanical thrombectomy procedures.^1^ A number of studies have shown that the use of BGCs in conjunction with stentriever devices is associated with improved clinical outcomes and procedural improvements, including shorter procedure times.^2–5^ The clinical improvements associated with BGC use are perceived to be a result of the temporary blockage of antegrade flow in the internal carotid artery (ICA), thereby achieving proximal flow control and reducing the risk of distal embolization.^6 7^ More recently, the combined use of contact aspiration with stentriever devices has become a popular first-line treatment strategy with several variations of the techniques being investigated including ARTS, CAPTIVE, SAVE, and SOLUMBRA.^8–11^ In a recent study by Bourcier *et al,* the authors established that there is no clinical or procedural benefit of the use of a BGC over a conventional guiding catheter when the combined stentriever+aspiration technique is used.^12^ This implies that the proximal flow control achieved by the BGC might not have an additional benefit beyond the effect of local aspiration through an aspiration catheter.

BGCs are commonly placed in the proximal ICA, as navigation of a relatively stiff BGC through the tortuous petrous and cavernous segments is challenging, and is associated with an increased risk of iatrogenic dissection.^13^ The middle cerebral artery (MCA)-M1 segment is the most common (58%–69%) primary occlusion site in large vessel occlusion strokes of the anterior circulation, with intracranial ICA occlusions comprising only 15%–22% of cases.^14–16^ In the treatment of MCA occlusions, BGCs achieve proximal flow control in the ICA; however, collateral inflow from the Circle of Willis (COW) via the posterior and anterior communicating arteries (PComm and AcoA) often ensures that antegrade flow still exists in the MCA. Therefore, in comparison to ICA occlusions the benefits of a BGC are likely reduced for MCA occlusions.

A new generation of ‘superbore’ aspiration devices with an inner lumen of 0.088 inches has recently emerged and early clinical evidence suggests that their use for thrombectomy in patients is safe and effective.^17 18^ These include the Millipede 088 (Perfuze, Galway, Ireland),^19^ the Zoom 88 (Imperative Care, Campbell, CA, USA),^18^ and the Ascender 088 (Route 92 Medical, San Mateo, CA, USA)^20^ catheters. Lally *et al* previously demonstrated that aspiration catheters achieve flow reversal at the tip of the catheter; however, the suction force reduces exponentially as the distance from the catheter tip increases, because fluid is drawn from both proximal and distal to the catheter tip.^21^ Increasing the lumen size of an aspiration catheter increases the aspiration force of the device,^22^ while the space-occupying effect of closely sizing the catheter outer diameter (OD) to the vessel inner diameter (ID) reduces the amount of proximal inflow. These effects combine to increase the distance from the catheter tip at which flow reversal can still be achieved.^21^ Therefore, the novel ‘superbore’ aspiration catheters may optimize local flow control and distal flow reversal, potentially resulting in improved revascularization outcomes while potentially obviating the need for BGC use.

The main purpose of this in vitro thrombectomy study was to (1) establish flow patterns in terms of both distal flow control and distal flow reversal using various aspiration catheter sizes, with and without the concomitant use of BGCs, and (2) evaluate the potential technical benefits of closely sizing the catheter-to-vessel size in terms of revascularization outcome and prevention of distal emboli.

## Methods

The data that support the findings of this study are available from the corresponding author on reasonable request. Approval for the study was granted by the National University of Ireland Galway Research Ethics Committee (19-DEC-11) and in accordance with the ethical standards of the Declaration of Helsinki. Human whole blood and platelets donations were obtained from the Irish Blood Transfusion Service and donors gave their informed consent for their donations to be used for research purposes.

### In vitro thrombectomy model

**Figure 1:**
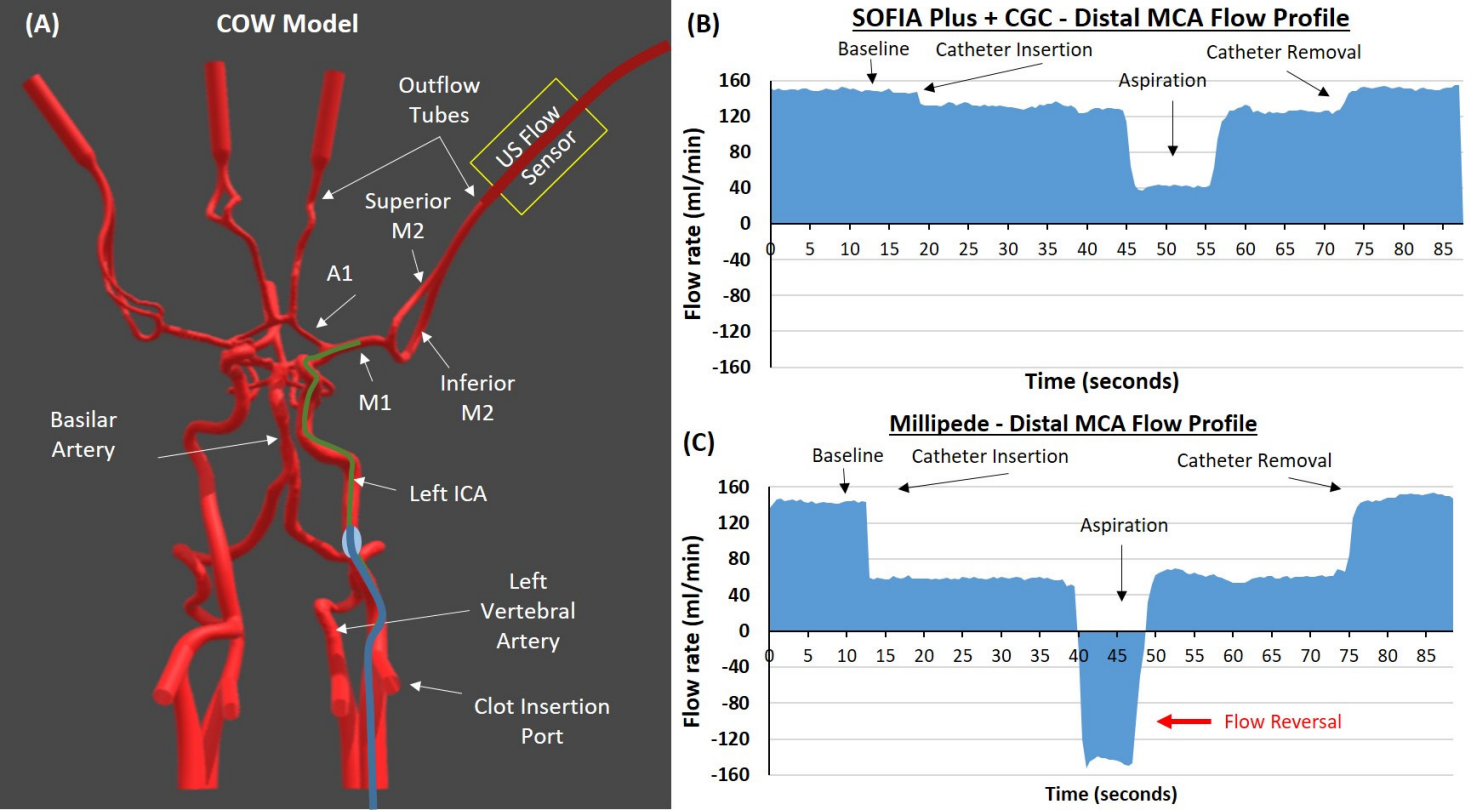
Placement of catheter and resulting flow profiles. (A) Circle of Willis (COW) model from the in vitro thrombectomy setup used to test flow profiles following catheter placement in the left middle cerebral artery (MCA). The ultrasonic flow sensor measures flow in the distal MCA. (B) Distal MCA flow profile of Sofia Plus+balloon guide catheter demonstrating the changes in flow on catheter insertion, during aspiration, and on removal of the catheter. (C) Distal MCA flow profile of Millipede 088 showing presence of flow reversal during aspiration. ICA, internal carotid artery.

The in vitro thrombectomy setup was described previously^19^ with two additions, namely flow sensors to measure real-time flow in the distal MCA and test sieves to capture distal emboli generated during the retrieval attempts. Flow was measured continuously through the MCA using ultrasonic flow sensors (Sonoflow CO.55/060, SONOTEC GmbH, Halle, Germany) placed approximately 20 cm distal to the MCA and catheter tip (figure 1A.). The diameter of the MCA-M1 segment was 3.1 mm, which is the mean diameter of the proximal MCA in humans.^23^ The other relevant vessel diameters of the COW model are as follow: proximal ICA=5.1 mm, distal ICA=4.3 mm, AcoA=2.5 mm, ACA=1.2 mm, and PComm=3.0 mm. Flow control was evaluated according to two mechanisms: (1) flow reduction, defined as the percentage reduction in MCA flow on introduction of the aspiration catheter, and (2) flow reversal, defined as retrograde flow on vacuum application to the catheter. The flow rate and direction (whether antegrade or retrograde) were recorded.

#### Catheters and setup for distal MCA flow control and flow reversal testing

Four aspiration catheters with increasing diameter were evaluated: ACE 60 and 64 (Penumbra), SOFIA Plus (MicroVention), and Millipede 088 (Perfuze). An 80 cm Super Arrow-Flex (Teleflex, Wayne, PA, USA) long sheath (ID=0.113 inches) was placed in the proximal ICA. The 6Fr aspiration devices (ACE 60, ACE 64, and SOFIA Plus) were investigated with both a Neuron Max Conventional Guide Catheter (CGC) (Penumbra) and a 9Fr Merci Balloon Guide Catheter (Stryker, Fremont, CA, USA) (table 1). Millipede 088 was not investigated with a BGC as there is not a commercially available BGC that can accommodate the larger 8F OD of Millipede 088. Where extra support was required for catheters to reach the target vessel, a Penumbra 3Max was used. In order to ensure that the same volume of aspiration was applied in each test, a 60 cc VacLok syringe (Merit Medical, Jordan, UT, USA) was used to supply a static vacuum, and aspiration was complete when the syringe filled.

**Table 1:** Catheter tip dimensions and setups investigated

Aspiration catheter	Internal diameter (inches (mm))	Outer diameter (inches (mm))	Tested with CGC	Tested with BCG
ACE 60	0.060 (1.52)	0.080 (2.03)	Yes	Yes
ACE 64	0.064 (1.63)	0.080 (2.03)	Yes	Yes
SOFIA Plus	0.070 (1.78)	0.083 (2.11)	Yes	Yes
Millipede 088	0.088 (2.24)	0.107 (2.65)	Yes	No

BGC, balloon guide catheter; CGC, conventional guide catheter.

#### UV particle flow pattern evaluation

For the purposes of visualizing the flow patterns within the COW on catheter insertion and during aspiration, green ultraviolet (UV) fluorescent particles (355–425 µm; Cospheric LLC, Goleta, CA, USA) were added to the test fluid. A magnetic stirrer was used to ensure a homogenous distribution of particles and a UV lamp was positioned directly over the COW model and used to cause fluorescence of the UV particles. The experiments were performed in a dark room and experiments were recorded using a high-definition camera (StreamCam; Logitech).

#### Clot retrieval testing

The technical performance of Millipede 088 was compared with the SOFIA Plus catheter, which is the largest of the competitor devices used in the flow control experiments, the SOFIA Plus was tested with both the CGC and BGC. All devices were inspected for kinking and patency prior to testing. Two clot analogs were created for clot retrieval testing (red blood cell-rich and fibrin/platelet-rich) as previously described.^19 24^ Clots were inserted into the proximal ICA via the clot insertion port, flow (720 mL/min) was started, and the clot analog travelled distally and lodged under dynamic flow conditions. Clot volume was optimized to mimic the clinical scenario; 10 mm long clots lodged in the M1 and 20 mm long clots lodged in the ICA.^19^ Following lodgment of the clot analog in the target vessel, one stack of four calibrated test sieves (Endecotts, London, UK) of decreasing aperture sizes (1000, 500, 250, and 50 µm) were placed in line with the five outflow tubes at the beginning of each retrieval test to catch all fragments that embolized during device navigation and clot retrieval. At the end of each retrieval test, each test sieve was examined under a microscope and the number of fragments captured in each sieve was recorded.

An aspiration pump (Dominant Flex; Medela AG, Baar, Switzerland) was used to supply a static vacuum (−700 mmHg) through the aspiration catheter for each attempt. Once the aspiration catheter was in contact with the clot, aspiration was applied until free flow was seen in the canister, or, if the clot corked the catheter tip, the catheter was withdrawn under aspiration into the BGC or CGC, depending on the setup. Ten replicates of each in vitro test were performed for each of the three setups (SOFIA Plus+CGC, SOFIA Plus+BGC, Millipede 088) in each location (ICA and MCA), totaling 60 tests for the study. Endpoints were first-, second-, and third-pass successful recanalization defined as complete removal of the clot from the cerebral vasculature. Complete revascularization was deemed unsuccessful if one or more large distal emboli (defined as >1000 µm) occurred during retrieval and were captured in the 1000 µm aperture test sieve.

#### Statistical analysis

All statistical correlations were assessed and graphs were generated using GraphPad Prism 8. The independent t-test was used to compare the means of two independent groups. A one-way ANOVA was used to compare means across all catheter types.

## Results

### Static MCA flow control: flow reduction following aspiration catheter insertion

**Figure 2:**
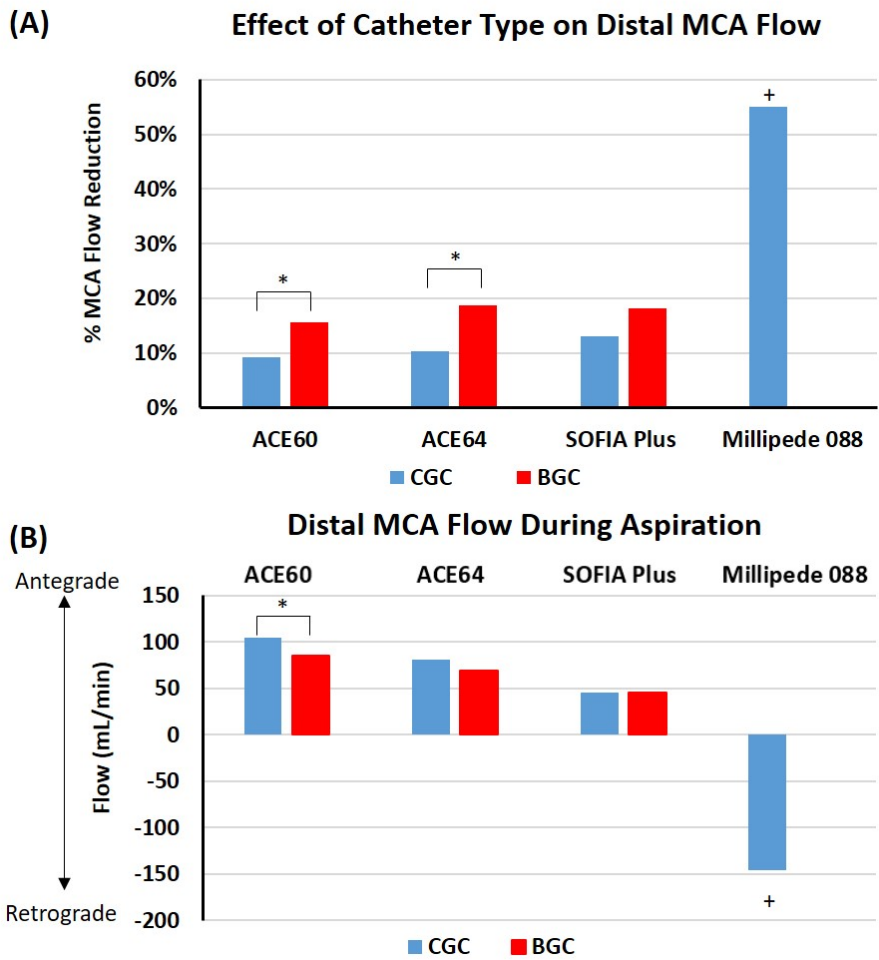
Effect of catheter size and use of a balloon guide catheter (BGC) on distal middle cerebral artery (MCA) flow control and on flow reversal during aspiration. (A) The percentage reduction in MCA flow of the aspiration catheters when used with a conventional guide catheter (CGC) (blue) or a BGC (red). (B) Distal MCA flow during aspiration with each catheter with both a CGC (blue) and BGC (red) during aspiration. CGC=blue, BGC=red, antegrade=forward flow, retrograde=flow reversal. *Denotes a significant difference between the CGC and BGC groups.+Denotes a significant difference between Millipede 088 and all other groups.

For each test, the aspiration catheter was positioned in the mid M1 segment of the MCA (figure 1A). Baseline flow rate in the MCA was 150±10 mL/min. Placement of each catheter in the M1 segment of the MCA resulted in a reduction in flow rate in the distal MCA as can be seen on the flow profiles for SOFIA+CGC (figure 1B) and Millipede 088 (figure 1C). The flow rate in the distal MCA decreased with increasing aspiration catheter OD when using a CGC (figure 2A). The use of a BGC led to a significantly greater reduction in the MCA flow rate than using a CGC with the ACE 60 (16% vs 9%, P=0.023*) (*denotes significance) and ACE 64 (19% vs 10%, P=0.007*) devices. The use of a BGC in combination with SOFIA Plus led to a numerical reduction in the MCA flow rate compared with the use of a CGC but this did not reach significance (18% vs 13%, P=0.086) (figure 2A).

The Millipede 088 resulted in a significantly greater reduction (55%) in distal MCA flow than all other aspiration catheters regardless of whether they were combined with a CGC or BGC (F(6,14) = 102, P<0.0001*) (figures 1C and 2A). The magnitude of flow reduction achieved by the Millipede 088 was three times greater than all other aspiration catheter and guide catheter combinations (figure 2A).

### Dynamic MCA flow control: flow reversal during aspiration

ACE 60, ACE 64, and SOFIA Plus were unable to cause flow reversal (retrograde) in the distal MCA during aspiration, meaning that there was still forward flow (antegrade) in the distal MCA during aspiration (figures 1B and 2B). Millipede 088 achieved a mean flow reversal of −146 mL/min in the distal MCA during aspiration (figures 1C and 2B). Millipede 088 had a significantly greater change from baseline MCA flow than all other aspiration catheters regardless of whether they were combined with a CGC or BGC (F(6,14) = 709, P<0.0001*) (figures 1C and 2B). The differences in flow patterns are highlighted in online supplemental video 1, which compares the flow rates before, during, and after aspiration with the SOFIA Plus+CGC, SOFIA Plus+BGC, and Millipede 088.

10.1136/neurintsurg-2021-017316.supp1 Supplementary video

#### Recanalization success and distal embolization

##### MCA occlusions

**Figure 3:**
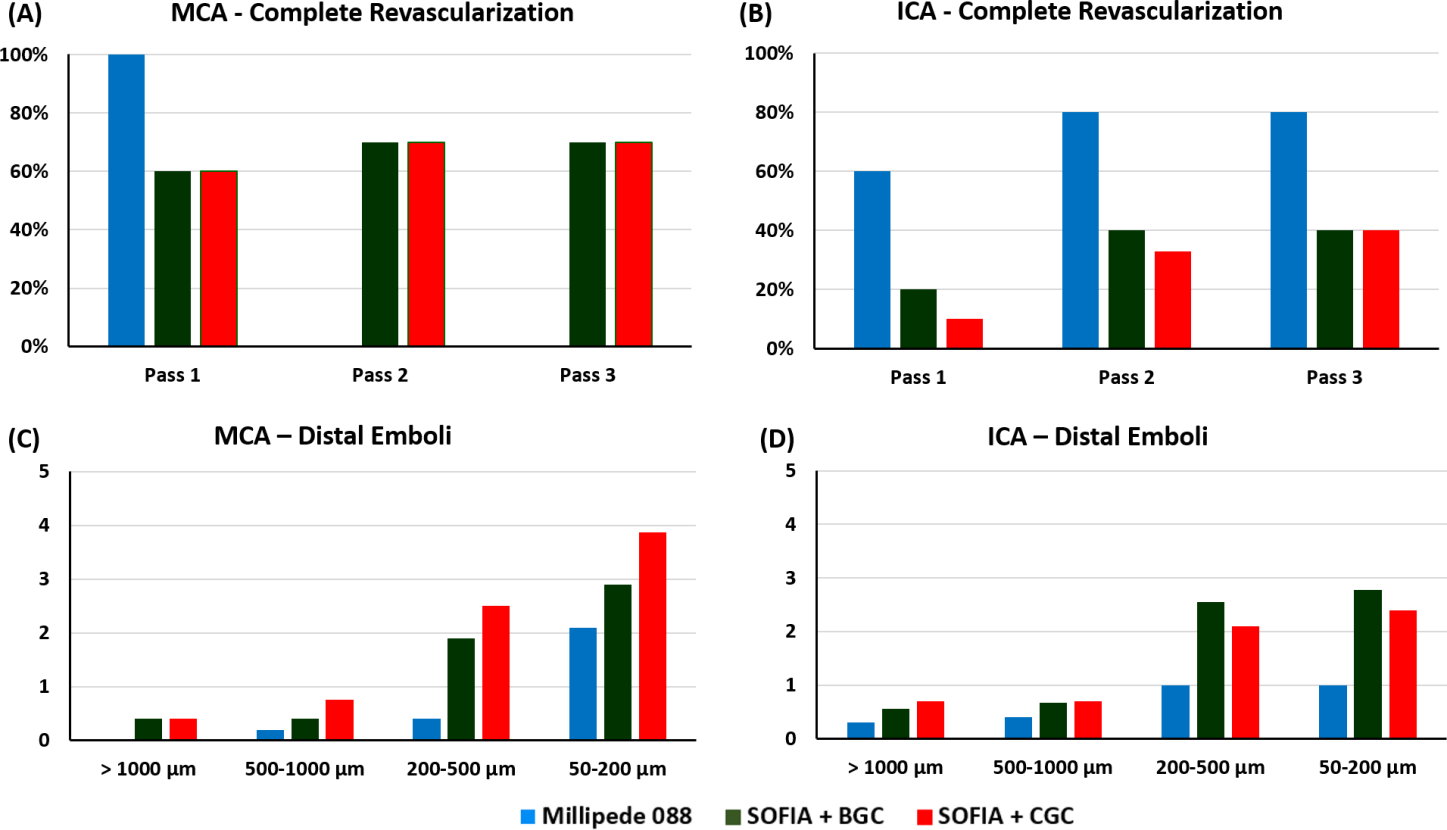
Results from the in vitro clot retrieval study. (A, B) Complete revascularization success rate after passes 1, 2, and 3 for middle cerebral artery (MCA) (A) and internal carotid artery (ICA) (B) occlusions. (C, D) The average number of distal emboli of each size (>1000, 500, 250, and 50 µm) generated during the clot retrieval attempts for MCA (C) and ICA (D) occlusions. Millipede 088=blue, SOFIA Plus+BGC (balloon guide catheter)=green, SOFIA Plus+CGC (conventional guide catheter)=red.

The Millipede 088 achieved 100% first-pass complete revascularization for MCA occlusions (figure 3A). The use of BGC with the SOFIA Plus did not lead to an improvement in the revascularization rate. The first-, second-, and third-pass success rates were 60%, 70%, and 70%, respectively, for both SOFIA Plus+CGC and the SOFIA Plus+BGC.

Overall Millipede 088 was associated with significantly fewer distal emboli of all sizes than SOFIA Plus+CGC and SOFIA Plus+BGC (P=0.014*) for MCA occlusions (figure 3C). The use of BGC with the SOFIA Plus led to a reduction in the number of distal emboli of all sizes when compared with the SOFIA Plus+CGC, though this did not reach statistical significance (P=0.356).

The benefit of Millipede 088 is demonstrated in online supplemental video 2. On aspiration, Millipede 088 completely ingests the clot in the MCA followed by the achievement of distal flow reversal in the MCA facilitating the evacuation of emboli distally and thereby achieving complete revascularization in one procedural pass. Conversely, on aspiration with the SOFIA Plus+CGC, the clot corked the aspiration catheter. The corked SOFIA Plus was then withdrawn under aspiration into the 8Fr sheath. As the catheter is corked, there is no local flow reversal in the MCA and consequently a fragment of clot occluding the superior M2 persists, requiring a second pass to achieve complete revascularization. These videos highlight the importance of complete clot ingestion and subsequent local and distal flow reversal in the target vessel.

10.1136/neurintsurg-2021-017316.supp2 Supplementary video

#### ICA occlusions

The use of BGC with the SOFIA Plus led to an improvement in the complete revascularization rates compared with SOFIA Plus+CGC in the first (20% vs 10%) and second (40% vs 33%) passes (figure 3B). The complete revascularization success rate was 40% in both the SOFIA Plus+BGC and the SOFIA Plus+CGC after the third pass. The Millipede 088 achieved superior first-, second-, and third-pass complete revascularization success rates of 60%, 80%, and 80%, respectively.

Millipede 088 was associated with significantly fewer distal emboli than SOFIA Plus+CGC and SOFIA Plus+BGC (P=0.015*) for ICA occlusions (figure 3D). The use of BGC with the SOFIA Plus did not significantly reduce the number of distal emboli of all sizes when compared with the SOFIA Plus+CGC (P=0.184).

## Discussion

This study assessed flow patterns in the distal MCA during in vitro thrombectomy procedures, and the results suggest that greater local flow control is achieved in the MCA with a ‘superbore’ catheter than with a 6Fr catheter paired with a BGC, potentially obviating the need for a BGC in MCA occlusions. Moreover, the increased surface area and consequential increased aspiration force of the ‘superbore’ Millipede 088 catheter causes flow reversal in the distal vasculature during aspiration resulting in improved revascularization rates and reduced numbers of procedure-related distal emboli. The use of a BGC with 6Fr catheters was associated with better revascularization rates for ICA occlusions but not MCA occlusions. These findings are clinically relevant as they suggest that the use of ‘superbore’ aspiration catheters for contact aspiration thrombectomy may achieve better revascularization outcomes and a reduced occurrence of distal emboli compared with the currently used 6Fr devices even when paired with a BGC.

The benefits of achieving flow arrest using BGCs during mechanical thrombectomy procedures has been highlighted previously^9 25^ and has been shown to be associated with improved recanalization and functional outcomes.^3^ However, the COW maintains antegrade flow in the MCA with the level of inflow from the COW being directly related to the size of the communicating arteries (PComm and AcoA) as well as the first segment of the anterior and posterior arteries which vary considerably among individuals.^26 27^ It has previously been suggested that closely sizing the OD of the catheter to the ID of the vessel reduces the antegrade flow in the target vessel, thereby achieving better local flow control and improving the technical performance of contact aspiration.^28^ This study confirms that placement of the ‘superbore’ Millipede 088 aspiration catheter in the MCA achieves a reduction in MCA flow that is three times greater than that achieved with the commonly used 6Fr devices tested, even when they are combined with a BGC. Therefore, for MCA occlusions flow control is better achieved through the placement of a catheter that has an OD close in size to the ID of the vessel than via proximal flow control with a BGC, potentially obviating the need for a BGC in the treatment of MCA occlusions. Removing the need for BGCs would remove BGC preparation time, and lead to a procedure-related cost saving for the patient as it can be replaced with a simple long 8F sheath.

Catheter size was also previously identified as an independent predictor of successful revascularization for aspiration thrombectomy.^29^ This study demonstrates that during aspiration the rate and direction of flow in the distal MCA is associated with the lumen size of the aspiration catheter. The ACE 60, ACE 64, and SOFIA Plus devices were unable to produce flow reversal (retrograde flow) in the distal MCA during aspiration even when paired with a BGC, meaning that there is still antegrade flow in the distal MCA during aspiration. The rate of antegrade flow decreased proportionally with increasing catheter ID from ACE 60 to SOFIA Plus. Contrastingly, Millipede 088 achieved significant flow reversal (retrograde flow) in the distal MCA during aspiration, which is highlighted in online supplemental video 1. The benefit of achieving retrograde flow in the distal MCA is that emboli that have traveled more distal than the site of the major occlusion may be drawn back into the aspiration catheter, thereby improving reperfusion. This could be key in achieving better first-pass effect rates, which is the ultimate goal of mechanical thrombectomy procedures.^30^

It is logical that the combined effects of reduced antegrade flow due to the larger OD and increased aspiration force due to the large ID of the ‘superbore’ Millipede 088 will increase the technical success of contact aspiration thrombectomy procedures compared with 6Fr devices, as we have shown previously.^19^ However, the influence of device size on local flow control and distal flow reversal, as well as the effect of concomitant use of a BGC with 6Fr devices and the number and size of procedure-related distal emboli, have not been previously investigated. This study demonstrates that Millipede 088 achieved superior recanalization success and reduced numbers of distal emboli when compared with SOFIA Plus+CGC and SOFIA Plus+BGC for both ICA and MCA occlusions. This result is attributable to the higher rate of clot ingestion with the Millipede 088 and the achievement of flow reversal in the MCA, thereby facilitating the evacuation of emboli that had traveled more distally in the vasculature. In agreement with the previous clinical study, we demonstrate that the use of a BGC had no impact on recanalization success in MCA occlusions^12^ and was associated with slightly fewer distal emboli than with a CGC. In ICA occlusions, the use of a BGC was associated with better first- and second-pass revascularization rates compared with the CGC. This highlights the benefit of a BGC over a CGC for ICA occlusions if using a 6Fr device that has a smaller catheter-to-vessel size, but the results obtained were still inferior to the Millipede 088 catheter.

This study has limitations. First, the in vitro thrombectomy model does not contain M3 and M4 vessels, meaning that the true effect of the distal emboli on revascularization outcome cannot be fully assessed. Second, this study only relates to the anterior circulation (and specifically to a model with a complete COW); however, it is plausible that the results should also be applicable to occlusions involving different anatomies including the posterior circulation. The use of BGCs is less common in the posterior circulation as one would need to occlude both vertebral arteries to achieve complete flow control. Therefore, closely matching the catheter-to-vessel size in the posterior circulation will likely also lead to better revascularization outcomes; this will be investigated in a later study. Third, newer 0.074 inch ID catheters are now used clinically; however, these catheters were not available for testing in this in vitro study.

## Conclusions

Two key behaviors are observed. First, closely matching the catheter size to the vessel size in the MCA allows the physician to control the degree of local flow on catheter insertion. Second, a 0.088 inch ID aspiration catheter enables significant flow reversal in the distal MCA during aspiration. The ultimate benefit of achieving retrograde flow in the distal MCA following full clot ingestion with Millipede 088 may be improved revascularization rates and reduced distal emboli compared with standard aspiration devices.

## Data Availability

Data are available from the corresponding author upon reasonable request.
